# Complex Carbohydrate Utilization by the Healthy Human Microbiome

**DOI:** 10.1371/journal.pone.0028742

**Published:** 2012-06-13

**Authors:** Brandi L. Cantarel, Vincent Lombard, Bernard Henrissat

**Affiliations:** 1 Institute for Genome Sciences, University of Maryland, School of Medicine, Baltimore, Maryland, United States of America; 2 Architecture et Fonction des Macromolécules Biologiques, Aix-Marseille Université, Centre National de la Recherche Scientifique, Marseille, France; Laurentian University, Canada

## Abstract

The various ecological habitats in the human body provide microbes a wide array of nutrient sources and survival challenges. Advances in technology such as DNA sequencing have allowed a deeper perspective into the molecular function of the human microbiota than has been achievable in the past. Here we aimed to examine the enzymes that cleave complex carbohydrates (CAZymes) in the human microbiome in order to determine (i) whether the CAZyme profiles of bacterial genomes are more similar within body sites or bacterial families and (ii) the sugar degradation and utilization capabilities of microbial communities inhabiting various human habitats. Upon examination of 493 bacterial references genomes from 12 human habitats, we found that sugar degradation capabilities of taxa are more similar to others in the same bacterial family than to those inhabiting the same habitat. Yet, the analysis of 520 metagenomic samples from five major body sites show that even when the community composition varies the CAZyme profiles are very similar within a body site, suggesting that the observed functional profile and microbial habitation have adapted to the local carbohydrate composition. When broad sugar utilization was compared within the five major body sites, the gastrointestinal track contained the highest potential for total sugar degradation, while dextran and peptidoglycan degradation were highest in oral and vaginal sites respectively. Our analysis suggests that the carbohydrate composition of each body site has a profound influence and probably constitutes one of the major driving forces that shapes the community composition and therefore the CAZyme profile of the local microbial communities, which in turn reflects the microbiome fitness to a body site.

## Introduction

Carbohydrates in the form of glycoconjugates, oligo- and polysaccharides represent one of the most diverse sets of molecules on Earth. An astounding diversity of carbohydrates is made possible by the different monosaccharide structures that can be assembled in a number of fashions to other sugar molecules, or to virtually any molecule of life, such as lipids, nucleic acids, proteins, antibiotics [1], [2], [3]. The resulting structural diversity is exploited by living organisms to perform with a high specificity a multitude of biological roles that can be assigned to broad categories such as structure and reserve, and intrinsic vs. extrinsic recognition [4]. Plants have evolved a particularly elaborate carbohydrate metabolism to synthesize a thick protective cell wall made of numerous polysaccharides and whose structure is resistant to enzymatic conversion. Carbohydrates also play an immense role in the host:microbiome interactions, such as providing nutrients to both host and flora, or as mediators that control the complex relationships between the two partners [5].

The selective assembly of glycoconjugates and complex carbohydrates is catalyzed by glycosyltransferases (GTs) while the deconstruction of the resulting structures is achieved by specific glycoside hydrolases (GHs) and polysaccharide lyases (PLs). Collectively these enzymes have been termed “Carbohydrate-active enzymes” (CAZymes), which are classified in a number of sequence-based families in the CAZy database (www.cazy.org) [6], [7], [8], [9], [10]. Because the number of protein folds is much smaller than the number of carbohydrate structures to build or break down, the sequence (hence structural) based families of CAZymes most frequently group together enzymes of differing substrate specificity, i.e. enzymes with different EC numbers [11]. One consequence is that it is difficult to predict the exact specificity of a CAZyme based on family membership. Despite this limitation, an inspection of the content of the families shows that a broad substrate category can often be associated to a CAZy family (Table S1) even if the precise specificity of each protein in the family is usually hard to predict reliably. Examination of the human genome reveals that it encodes 97 GHs, with only 17 enzymes to breakdown carbohydrate nutrients, nine of which are not yet fully characterized (Table S2). This is a tiny number compared to some of the gut bacteria such as *Bacteroides thetaiotaomicron* [12], which, alone, encodes over 260 GHs (see www.cazy.org/b135.html).

It has been estimated that the human body is inhabited by 10^14^ microbes, with the human gut thought to contain about 7000 different strains [13]. The Human Microbiome Project (HMP) consortium has collected and analyzed Whole Genome Shotgun (WGS) sequence information from microbial communities isolated from five major body sites inside and on the surface of the human body (Gut, Airways, Oral, Urogenital and Skin) [14]. As a collaborative analysis with the HMP consortium, we have compared the prevalence and abundances of CAZymes from 148 subjects collected in these 5 major body sites, yielding 520 samples (www.hmpdacc.org/HMPGOI). To determine the predictive power of taxonomic community structure to reconstruct the functional repertoire of a microbial community, we have built functional profiles for these samples, using their 16 S taxonomic profile and gene content based on reference genomes. Lastly, we compared the gene content of 493 reference genomes isolated from the human body in order to determine whether human-associated bacterial genomes, some of which being part of the consortium project [15], inhabiting a particular habitat had gene content more similar than microbes in the same taxonomic family. Examination of the family prevalence and of the broad carbohydrate categories shows that the CAZyme profiles of each body site are different, and adapted to the particular carbohydrate composition of the body site.

## Results

### Comparison of Reference Genomes Indicates Gene Content by Taxonomic Family

**Figure 1:**
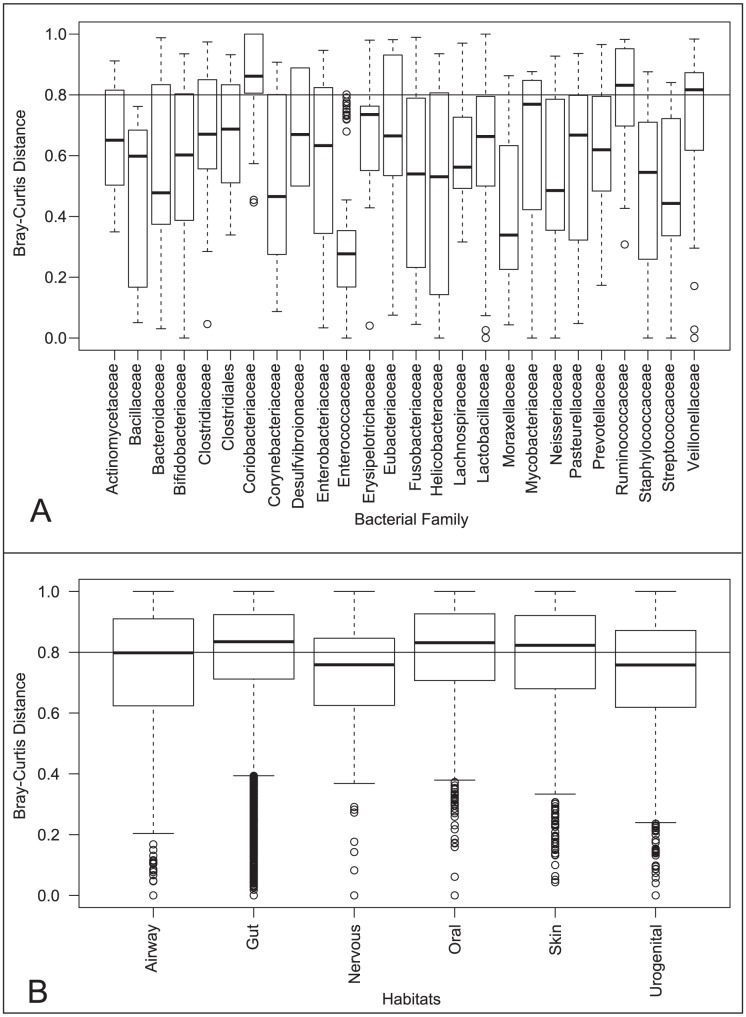
Comparison of Reference Genomes. Bray-Curtis distances were calculated, using the relative abundance of gene families normalized by housekeeping genes GT28 and GT51, between the 493 human associated bacterial genomes using the ecodist library in R and compared within genomes in the same bacterial family (A) or the same general body site (B).

Microbes living inside and on the surface of the human body have employed horizontal gene transfer and gene duplication to gain functions [16], [17], [18], [19], particularly in CAZymes [20], [21] to adapt to constraints of their particular environment. Therefore, we wanted to assess the similarity of the CAZyme repertoire in human associated bacterial genomes in the same taxonomic family compared to that in the same body site (Table S3). We have decided to compare the complex carbohydrate utilization abilities of 493 human associated bacterial genomes, by determining their profiles in GH and PL enzymes which both cleave glycosidic bonds, even though they use different chemical mechanisms. When we compare a Bray-Curtis distance between samples, the distribution of distances is lower among most bacterial families (Figure 1A) compared body sites (Figure 1B). However, some bacterial families, such as *Coribacteriaceae*, *Ruminococcaceae*, and *Veillonellaceae*, are more diverse in their CAZyme profiles and have mean distances similar to the genomes of bacteria inhabiting the same body site.

When we compare the mean number of sugar-cleaving enzymes, GHs and PLs, encoded by genomes within a bacterial family, the abundance of these enzymes varies greatly (Table S4). For example in the family *Bacillaceae* the mean number of GHs is 25, with a standard deviation of 3.3. In contrast, *Clostridiaceae* has on average 56 GHs, yet the range of that is much broader as the standard deviation is about 79. Therefore, even within a bacterial family, the copy number of GHs and PLs can have a wide distribution, making the prediction of the gene content in an unknown genome difficult.

### Relative Abundance of CAZymes in Major Body Sites

**Figure 2:**
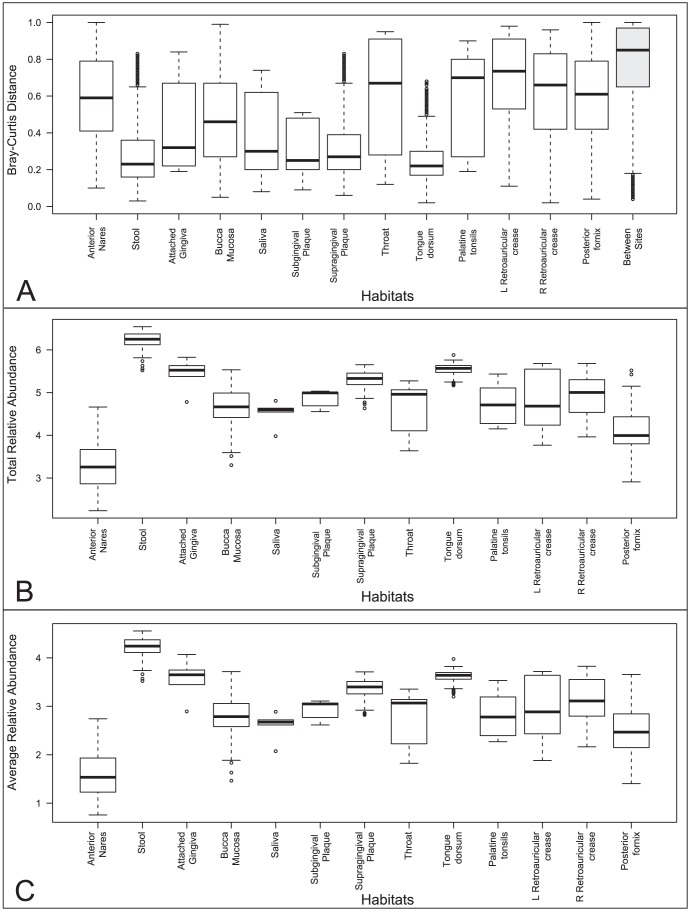
Comparison of Relative Abundance of Healthy Human Microbiome Samples. (A) Bray-Curtis distances were calculated on relative gene abundances between samples and grouped by same body habitat (white boxes) or distinct habitat (grey box). (B) Total relative abundances are the results of adding the normalized relative abundances of each sample. (C) Average relative abundances are the total relative abundance divided by the total number of CAZy families in each sample.

To examine microbial communities in the human body, we annotated CAZymes in the reads and contigs publically available as part of the HMP Consortium project, HMIWGS dataset [14], which represent the largest collection of WGS sequence from the human microbiome. In order to assess the CAZyme potential of these 520 microbial communities collected from five major sites inside and on the surface of the human body (Table S5), we calculated the relative abundance of CAZymes on the metagenomic reads, in the 113 GHs and 19 PLs families that were known at the time of this analysis, normalized on the housekeeping genes GT51 and GT28, which have more stable copy numbers in reference genomes (Table S4). Analysis of CAZyme profiles using the Bray-Curtis distance metric (Figure 2A) showed that samples in the same body habitat (white boxes) are more similar to each other than those from distinct habitats (grey box). The body site with the highest total abundance of CAZymes is the gastrointestinal tract followed by the various oral samples (Figure 2B). These digestive body sites, on average, also have greater abundances of individual CAZy families represented (Figure 2C). Samples in the buccal mucosa and anterior nares at first glance appear to be poor in these enzymes, but upon further examination, these samples along with retroauricular crease are those with the highest amount of human sequence contamination, which translates into much lower sequence coverage [14].We identified 81 protein families that exhibited statistically significant (p-value <0.05) abundances in pair-wise comparisons among samples in each major body site (Table S6). Two enzyme families, GH94 (cellobiose, cellodextrin and chitobiose phosphorylases) and GH30 (β-1,6-glucanase, β-xylosidase, β-D-fucosidase, β-glucosidase and β-1,6-galactanase), are over-abundant in stool compared to the other four major body sites. Family GH19, which comprises enzymes involved in the breakdown of animal carbohydrates and fungal cell walls, is under-represented in vaginal samples compared to other sites. When we compare digestive body sites (Oral and GI) to non-digestive sites (Vaginal, Skin and Airways), we identify six families over-represented in digestive sites (Table 1), four of which are involved in plant (GH53 and GH94) and algal (GH117 and GH86) cell wall degradation.

**Table 1:** CAZymes statistically significant with differences in relative abundance between digestive site samples compared to non-digestive site samples.

	Digestive Site Samples		Other Site Samples		
Family	Mean Relative Abundance	Std Error	Mean Relative Abundance	Std Error	P-Value
GH53	1327.7	143.7	947.8	209.3	0.04
GH116	634.2	84.3	401.6	102.2	0.04
GH86	2.5	0.8	0.3	0.1	0.05
GH94	1354.6	173.3	640.2	167.7	0.01
GH117	83.9	21.2	38.9	21.1	0.02
GH35	5975.9	396.5	4790.4	642.5	0.05
GH82	0.7	0.3	0.0	0.0	0.00

### Differential Prevalence of CAZymes

**Figure 3:**
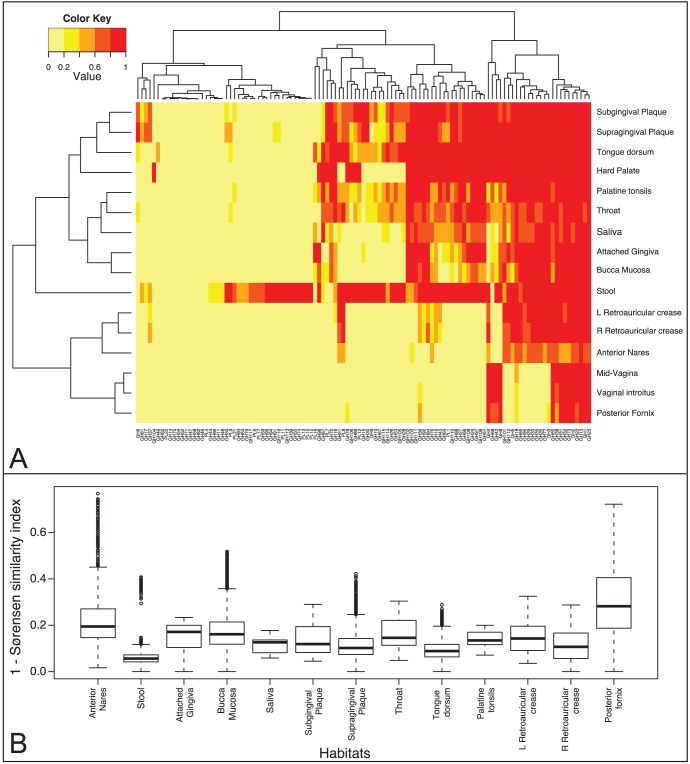
Comparison of Prevalence of Healthy Human Microbiome Samples. (A) Heatmap of genes prevalence per body site using a heat color scheme (yellow to red), indicating low to high prevalence. (B) Gene repertoire distance as calculated by 1 - Sørensen’s similarity coefficient, between samples originating from the same body habitat. Higher distances indicate a lower number of proportionally shared genes between any two samples from the same body site.

In addition to comparing the relative abundance of enzymes in body sites, we also compared the presence of GHs and PLs on metagenomic assemblies (www.hmpdacc.org/HMGI) [14]. The prevalence of each gene family was calculated as the fraction of the samples containing the family per body site. Hierarchical clustering by gene family prevalence per body site shows similarities among habitats regardless of differences in sequence coverage seen between sites with high and low human genomic contamination. For example, by examining the enzymes present in oral samples, we discovered that these habitats (Buccal mucosa, supra- and super- gingival plaque, tonsils and tongue) are much more similar to each other than gastrointestinal, urogenital and nasal and skin habitats (Figure 3A). Core families, represented in >95% of samples, are involved in energy production (GH32, GH31, GH38, GH1, GH13) and peptidoglycan breakdown (GH73, GH25, GH23). There are a number of families that are uniquely prevalent to stool which are most likely involved in the digestion of animal (GH79, GH99, PL6, PL13), fungal (GH55, GH64, GH113), plant (GH39, GH74, GH91, GH93, GH94, PL10, PL11) and algal (GH117, PL17, PL15) polysaccharides. The latter results shows that algal polysaccharide breakdown is present in the individuals that were investigated by the HMP consortium, an observation that contrasts with a report that porphyranases and agarases are frequent in the Japanese population and are absent in metagenome data from North American individuals [22]. Our finding of families GH50 (agarases), GH86 (agarases) and GH117 (neoagarooligosaccharide hydrolases) in most oral and stool samples suggests that agarose digestion is largely present in the North American population. Additionally, we compared the gene content of samples within each body site using the Sørensen similarity index, which is a calculation of the number of genes present in two samples compared to the total number of gene families. The genes present in stools samples are the most conserved, whereas vaginal habitats exhibit higher diversity (Figure 3B). These results are consistent with other studies of this dataset based on taxonomy [23] and KEGG Orthologs (KOs) groups [24]; in these studies and in a publication by Ravel and colleagues, the posterior fornix is shown to have the most diverse repertoire of functional capability [23], [24] and a few distinct community types [23], [25].

Using the publically available taxonomic annotations of these genes [14] (HMGI), we were able to compare the CAZyme abundance from a bacterial family by body site to the reference genome results. Because of the taxonomic diversity of most human habitats, metagenomic sequences, even assembled, are often short fragments, since we are unable to reach sequence saturation. Consequently, our ability to detect CAZymes from particular taxa is reduced compared to the single genomes, which will be a diminishing concern as sequence generation increases. For some bacterial families such as *Corynebacteriaceae* relative abundance of GHs and PLs is very similar (7.9±1.5 in vaginal samples vs. 9.8 in oral samples). These taxa are estimated to have 17±7.8 GHs and PLs per genome (Table S3), suggesting similar copies of GHs and PLs per genome. However for other families like Enterobacteriaceae (44±18.9 GH+PLs in reference genomes), there is a 10 fold difference between the number of GHs and PLs in stool compared to oral site (12.3±2.7 vs 1±0.36), possibly reflecting either a selection of genomes with a high number of GHs and PLs in stool.

**Figure 4:**
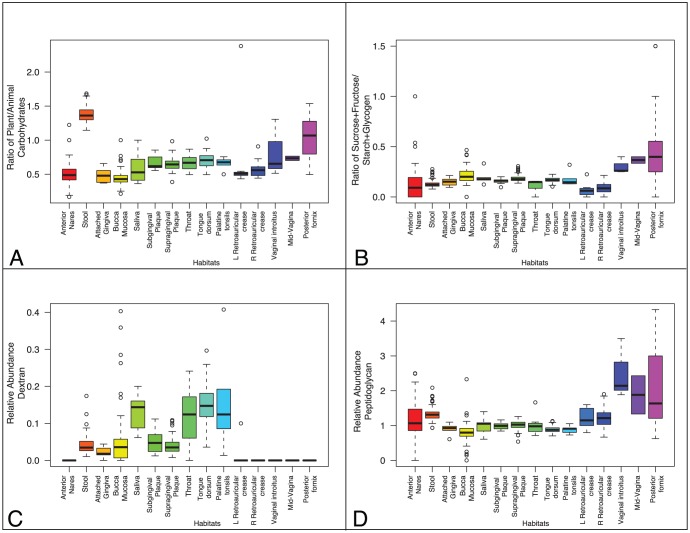
Sugar Utilization Potential of Microbiome Samples. (A) The ratio of the number of proteins that hydrolyze plant cell wall carbohydrates to proteins that hydrolyze animal carbohydrates. (B) The ratio of the number of proteins that hydrolyze sucrose or fructan to proteins that hydrolyze starch or glycogen. (C) Relative abundance of the proteins that hydrolyze dextran. (D) Relative abundance of the proteins that hydrolyze peptidoglycan.

In order to get an estimate of the enzymatic capabilities and possible pathways for each body site, we have assigned each CAZy family to their broad substrate categories, namely animal, plant cell wall and fungal carbohydrates, starch and glycogen, dextran, peptidoglycan and sucrose and fructans (Table S1). Although these broad substrate categories do not allow identification of the precise targeted carbohydrates, except for family GH68, which is involved in converting sucrose into biofilms made of fructose polymers (fructan), they allowed us to compare the broad sugar utilization of each body site based on the average of samples within that body site. Four major carbohydrate utilization profiles emerge (Figure 4): (a) as expected, anterior nares and retroauricular crease environments have relatively low capacity for the degradation of the sugars in these categories (b) the microbes in the vagina contain a higher proportion of enzymes involved in sucrose cleavage and polymerization to fructans than the other body sites, potentially for biofilm formation, (c) oral communities have a balanced utilization of these nutrient derived carbohydrates in each subsite, including dextran, which appears to be unique to oral site, suggesting GH70, GH66 and GH87 as potential markers for plaque formation and (d) stool communities have the highest capacity and variety of plant cell wall polysaccharide-cleaving enzymes. CAZy families GH4, GH68, GH42 and GH8, appear to be highly prevalent in the oral, the supra and subgingival plaque sites in particular, and the vagina (Figure 3A). Interestingly the GH8 and GH68 enzymes are probably involved in polysaccharidic biofilm synthesis.

The human gut microbiota encodes a huge diversity of enzymes for the digestion of all components of plant cell wall polysaccharides including cellulose. Turnbaugh et al [26] have shown that the distal gut microbiota of humans also encodes dockerin-containing cellulolytic enzymes that indicate the presence of cellulosomes (multienzymatic complexes of plant cell wall digesting enzymes assembled on a large scaffolding protein) [27], [28]. The present HMP analysis confirms the presence of dockerin-containing proteins in the GI and identifies several in the oral, nasal and vaginal samples (Table S5), which is not surprising given their presence in human-associated bacterial reference genomes. In non-digestive sites, however, these dockerin domains may have another role than attaching cellulases to form a cellulosome.

### Predicting the CAZome from Taxonomic Profiling

While the cost of next-generation sequencing continues to decrease, studies of hundreds of samples with metagenomic sequencing might be prohibitory for the average researcher whom might be tempted to economize by using 16 S rRNA sequencing and reference genome gene content to predict a functional profile. However, it is unclear whether variation in bacterial communities translates to diversity in functional capability and whether these distinctions can be predicted by community taxonomic profiling. For example, at the genus-level assignment, the posterior fornix, appears to be the least diverse because of dominance by Lactobacillus, however across the population, there appears to be several distinct community types [25]. Our CAZyme analysis suggests that these distinct communities in the posterior fornix might translate into distinct functional types, a result consistent with pathway analysis of these same communities [24]. In contrast, stool has many more genera and therefore is the more taxonomically diverse, yet is the most functionally conserved. Using these 16 S taxonomic profiles and the publicly available reference genomes, we tried to reconstruct the CAZy profile of a select group of samples where 16 S and WGS data were available from the same specimen. In general, we found that the predicted profiles do not resemble very well the actual metagenomic profiles (Figure S1). First, based on relative abundances, we found that the discrepancy between the 16 S predicted and the actual WGS CAZy profile is greater in stool than in oral or vaginal body sites (Figure S1A). Similarly, the gene repertoires in oral and stool communities are also underestimated using 16 S rRNA and reference genomes (Figure S1B; red, blue and green circles). Interestingly, however, the posterior fornix shows the opposite trend, likely due to the higher coverage by reference genomes (Figure S1B; purple circles). These results could indicate (i) there are differences in gene content between organisms in the same genus, which cannot be seen in 16 S because of gene diversity within a species or strain, and/or (ii) we lack reference genomes for some genera. For example, in stool, on average 13% (60% in some samples) of the 16 S sequences classify to a genus without a sequenced reference genome in that genus, representing about 17% of all surveyed genera. Additionally, some of the genes that we identified as differentially abundant between body sites would not have been predicted as being present or under-abundant, such as GH6 in the oral sites and GH94 or GH48 in the GI (Figure S1C). In addition, the importance of some proteins would have been over-predicted, such as GH32 in the anterior nares and posterior fornix. Therefore, while 16 S sequencing is a good survey of taxonomic diversity, without more reference genomes, it does not serve as a good proxy for determining the precise carbohydrate utilization capabilities of digestive microbial communities.

## Discussion

The human genome encodes less than 20 enzymes for the digestion of complex carbohydrates, mostly plant reserve carbohydrates (sucrose and starch) and lactose. However, the cell walls of plants represent an enormous nutrient source, yet highly variable in terms of amount, diversity and botanical source [29], [30]. These carbohydrates are chemically and structurally highly complex, and are arranged in a three-dimensional network that has evolved to be intrinsically resistant to enzymatic breakdown [31]. Thus high molecular weight crystalline cellulose microfibrils are inter-twinned with hemicelluloses and pectins, which are a whole range of homo- and heteropolysaccharides composed of dozens of different monosaccharide units linked in a multitude of ways. Ester substituents or non-carbohydrate polymers, such as lignin, proteins, cutin and suberin, add a further layer of complexity. As a result, a single vegetable contains hundreds of different bonds that need to be cleaved in order to unlock the assimilable carbon of the cell wall constituents. Considering the considerable variations (in composition and in microscopic structure) in the cell walls of the vegetables and fruits in the human diet, the digestive enzymes face a huge number of different substrates. Since these enzymes are absent from the human genome, humans rely on the microbiota inhabiting the digestive track to utilize these complex plant polysaccharides [13]. The microbiota must adapt rapidly to environmental cues to determine which enzymes are necessary to metabolize the plant cell wall structures in each meal.

Digestion starts in the oral cavity, where this study suggests that microbes have a hitherto underestimated large range of enzymes to initiate plant polysaccharide breakdown as indicated by the presence of cellulases (GH6), hemicellulases (GH26) and pectin hydrolases (GH28 and GH43). Additionally, microbes in the oral cavity also initiate the processing of ‘easy’ plant carbohydrates, such as sucrose and starch, which can be converted into biofilms (dextran, fructans) that secure long-term residence to the bacteria in the oral sphere. When we compare the oral sites to the gut, the starch and glycogen utilization appears much reduced in stool, suggesting specialization in digestion, whereby most of these sugars are likely degraded by human salivary amylase and oral flora and taken up in the small intestine by the host [32]. The starch molecules that do reach the distal gut are particularly difficult to hydrolyze and are known as “resistant” starch [33] and are considered a component of dietary fibers.

Upon examination of the mechanisms important for plant cell-wall degradation, we found that non-reducing end acting cellobiohydrolases (CAZy family GH6) appear specific to the oral cavity while reducing end acting cellobiohydrolases (family GH48) are specific to the gut. The two types of cellobiohydrolases have been found to digest cellulose in a synergistic manner when acting together or when acting with endoglucanases [34]. The current paradigm is that cellobiohydrolases are the essential cellulases because they can deliver soluble cellobiose from polymeric cellulose in a single step, and the role of the endoglucanases is to provide chain ends to the cellobiohydrolases [31]. The GH6 genes found in the oral cavity, were highly prevalent in supragingival plaques samples and are primarily attributed to the genera *Capnocytophaga*. The GH48 genes, found in stool, are mostly in unknown taxon, although likely in some species of the *Firmicutes* genus *Ruminococcus*. However, there is no reference genome in this genus with annotated GH48 genes. Thus far, the GH6 family of enzymes has not been identified in any animal gut sample [35], [36], [37], [38], [39], suggesting that this enzyme specificity is perhaps driven by environmental factors.

Based on this study, digestion in the gut appears highly specialized for the digestion of complex carbohydrates. Since the other body sites are unlikely to be exposed to plant carbohydrates for a significant length/amount, the plant carbohydrate utilization is likely the most prominent factor to explain the great divide observed in the WSG metagenomic data between the digestive tract and the other body sites. In the gut, the proportion of genes that hydrolyze plant cell wall is greater than the genes that hydrolyze animal carbohydrates, probably reflecting the greater carbohydrate diversity elaborated by plants compared to that of animals. In almost every carbohydrate category, the gut microbiota has the highest ability to degrade these carbohydrates. The distal gut appears to have all the necessary enzymes for plant polysaccharide digestion with the puzzling exception of GH6 cellulases, suggesting that these enzymes have not been selected to breakdown cellulose substrates by anaerobic animal gut bacteria, while they are common in soil bacteria and fungi that decay plant cell walls. In conclusion, two major trends emerge. First the functional profile of the collective microbial community is more similar within a body site than between sites, despite variation of taxonomic profiles. This means that there is a specialization of the flora at each body site and that it is clearly detectable by metagenomic sequencing, suggesting that metagenomic sequencing is able to trace the functional adaptation to the carbohydrates that prevail in a given body site. Second, while broad predictions of the global number of CAZymes could possibly be made due to the overwhelming number of GHs and PLs encoded by Bacteroidetes compared to Firmicutes, the present results show that we are unable to predict the actual CAZyme profile at each body site due to an insufficient number of reference genomes. However, without a better knowledge of the precise substrate specificity of the enzyme families showing expansion/reduction, there seems to be little correlation between the functional capability and taxonomic family. These results suggest the exact functional profile of CAZymes by body site is not currently predictable given genera abundances. Whilst the current efforts aimed at sequencing more reference genomes will sooner or later allow a finer prediction, the precise functional CAZyme profiling will also require coupling metagenomic analyses to structural genomics initiatives and to high-throughput biochemical and other functional assays of CAZymes [40].

## Methods

### Reference Genome Annotations

In order to compare the gene content of reference genomes isolated from human body sites, protein sequences files from human associated genomes (Table S3) were downloaded from Genbank [41]. CAZyme protein family assignments were determined for each reference genome using a semi-automated pipeline [6] and normalized by the number of glycosyltransferases from families GT51 and GT28, as these are found to be stable in bacterial genomes (Table S4). Bray-Curtis distances were calculated between samples using the ecodist library in R. Distances were compared within each bacterial family and within each body site as separate analyses.

### Metagenomic Read Genome Annotations

Pre-human screened, trimmed and quality-filtered Illumina shotgun metagenomic reads (HMIWGS build 1.0) [14] were downloaded from http://hmpdacc.org/HMIWGS. Carbohydrate active enzyme annotations were performed on each read using MBLASTX (http://www.multicorewareinc.com/), with default parameters and against proteins in the CAZy database [6]. Relative abundances were calculated based on the matches and sequence length based on methods developed by Abubucker et al. [24] and normalized by dividing the relative count by the number of GT28 and GT51 relative counts. Bray-Curtis distances were calculated between samples using the ecodist library in R. Distances were compared between all samples and divided into same sample and distinct comparisons. Total relative abundances are the results of adding the normalized relative abundances of each sample. Average relative abundances are the total relative abundance divided by the total number of CAZy families in each sample. Gene families with statistically significant differential abundance between body sites were determined using METASTATS [42].

### Metagenomic Contig Genome Annotations

Assemblies from pre-human screened, trimmed and quality-filtered Illumina shotgun metagenomic reads, HMASM build 1.0 [14], were downloaded from http://hmpdacc.org/HMASM. Functional annotations were performed by BLASTX searches against proteins in the CAZy database [6], using thresholds: E-value <1e−6 and bits/position >1, and normalized by the abundance of reads assigned to families GT51 and GT28. Hierarchical clustering was performed for these genomes, using the complete method, by the matrix of Euclidean distances between genomes based on the normalized counts of GH and PL protein families. The prevalence of a gene was determined by calculating the fraction of the samples within each body site where that gene was present, at any abundance level. Sørensen’s similarity coefficient [43] was used to determine the similarity of the GH/PL profile between the samples in each body site. Based on the substrates for each GH and PL family (Table S3), the sugar utilization of each sample was estimated by summing the relative abundance of genes, which act on each substrate. The sugar utilization of each body site is an average of sugar utilization of each sample.

### Inferred Metagenomic CAZy Profiles

Phylotype information for samples, with corresponding 16 S rRNA profiles, was downloaded from http://hmpdacc.org/HMPOC [14]. The inferred metagenomic CAZy profiles were determined by using these taxonomic relative abundance tables for each sample. For each CAZy family in a sample, the profile was calculated by (i) determining the average number of that family for all reference genomes for each genera, (ii) multiplying that average by the fraction of the sample in each genera, (iii) summing each genera’s inferred contribution to each family and (iv) normalizing each sum per family by housekeeping glycosyltransferases from families GT51 and GT28. Hierarchical clustering is calculated for these genomes, using the complete method, by the matrix of Euclidean distances between genomes based on the normalized counts of GH and PL protein families, since a Bray-Curtis distance calculation is less sensitive to differences of scale. These profiles were then compared to abundances based on reads. Fraction of families conserved was calculated by the number of families presented in the WGS metagenomic or 16 S inferred profile divided by the total number of families observed whether they be inferred or observed.

## Supporting Information

Figure S1 **Inferred metagenomic profile comparison.** CAZy profiles were inferred from 16 S profile and reference genome gene content. (A) Euclidean distance between inferred (16 S) and WGS CAZy profile. (B) Comparison of Family membership as calculated by the fraction of CAZy families in both profiles found in the WGS profile (y-axis) and 16 S profile (x-axis). Circles colored by body site: Stool (red), Tongue Dorsum (blue), Supragingival plaque (green) and posterior fornix (purple). (C) Heatmap of inferred CAZy gene family abundances per body site using a heat color scheme (yellow to red), indicating low to high abundance.
(EPS)

Table S1 **Broad Substrate Categories of CAZy Families.**
(DOCX)

Table S2 **Human Digestive Enzymes.**
(DOCX)

Table S3 **Bacterial Genomes by Body Site.**
(DOCX)

Table S4 **Statistics of CAZy Genes per Genomes by Bacterial Family.**
(DOCX)

Table S5 **Metagenomic Samples by Body Site.**
(DOCX)

Table S6 **CAZymes with Statistically Significant Differences in Relative Abundant between Body Sites.**
(DOCX)
