# Supplementary material for: Complex Carbohydrate Utilization by the Healthy Human Microbiome
Source: PLoS One. 2012 Jun 13;7(6):e28742. doi: 10.1371/journal.pone.0028742 (PMC3374616; doi:10.1371/journal.pone.0028742)
Supplement: Table S1 — Broad Substrate Categories of CAZy Families. (DOCX) [file pone.0028742.s002.docx]

Table S1. Broad Substrate Categories of CAZy Families*.

| Broad Substrate | CAZy Families |
| --- | --- |
| Plant Cell Wall Carbohydrates | GH1; GH2; GH3; GH4; GH5; GH8; GH9; GH11; GH12; GH15; GH16; GH17; GH26; GH27; GH28; GH29; GH36; GH39; GH43; GH44; GH48; GH51; GH53; GH55; GH67; GH74; GH78; GH93; GH94; GH95; GH115; GH117; GH121; PL1; PL2; PL6; PL7; PL9; PL11; PL15; PL22 |
| Animal Carbohydrates | GH1; GH2; GH3; GH4; GH18; GH19; GH20; GH29; GH33; GH38; GH58; GH79; GH84; GH85; GH88; GH89; GH92; GH95; GH98; GH99; GH101; GH105; GH109; GH110; GH113; PL6; PL8; PL12; PL13; PL21 |
| Peptidoglycan | GH23; GH24; GH25; GH73; GH102; GH103; GH104; GH108 |
| Starch/Glycogen | GH13; GH15; GH57; GH77 |
| Sucrose/Fructans | GH32; GH68; GH70; GH91 |
| Fungal Carbohydrates | GH5; GH8; GH16; GH18; GH19; GH20; GH55; GH64; GH71; GH81 |
| Dextran | GH66; GH70; GH87 |

*If a family contains enzymes that act on two different substrate categories, it appears in each category
