# Supplementary material for: Complex Carbohydrate Utilization by the Healthy Human Microbiome
Source: PLoS One. 2012 Jun 13;7(6):e28742. doi: 10.1371/journal.pone.0028742 (PMC3374616; doi:10.1371/journal.pone.0028742)
Supplement: Table S2 — Human Digestive Enzymes. (DOCX) [file pone.0028742.s003.docx]

Table S2. Human Digestive Enzymes*

| **Known intestinal sugar-cleaving enzymes** | **Accession** | **CAZy family** | **Substrate** |
| --- | --- | --- | --- |
| Alpha-amylase 1 (Amy1) | AAH63129 | GH13 | starch |
| Pancreatic alpha-amylase (Amy2A) | AAA51724 | GH13 | starch |
| Alpha-amylase 2B (Amy2B) | AAA35525 | GH13 | starch |
| Lactase-phlorizin hydrolase | CAA30801 | GH1 | lactose |
| Maltase-glucoamylase, intestinal | AAP21875 | GH31 | maltose (from starch) |
| Sucrase-isomaltase, intestinal | AAT18166 | GH31 | sucrose, isomaltose |
| Trehalase | BAA24381 | GH37 | trehalose |
| Neutral alpha-glucosidase C | AAN74756 | GH31 | maltose (from starch) |
|  |  |  |  |
| **Possible candidates for additional digestive sugar-cleaving enzymes** | **Accession** | **CAZy family** | **substrate** |
| Uncharacterized family 31 glucosidase KIAA1161 protein | AAH70098 | GH31 | unknown |
| Chitotriosidase-1 CHIT1 (gastric expression) | CAC37767 | GH18 | chitin |
| Acidic mammalian chitinase (gastric expression) | AAG60019 | GH18 | chitin |
| Klotho-like protein | BAC56857 | GH1 | unknown |
| Lactase-like protein | AAQ89091 | GH1 | unknown; missing catalytic residue |
| FLJ90269 protein | AAQ88861 | GH35 (distant) | unknown |
| Beta-galactosidase-1-like protein 3 | AAH11001 | GH35 | unknown |
| endoglucanase-like protein | BAC04648 | GH9 | unknown |
| maltase-glucoamylase-like protein FLJ16351 | BAD18495 | GH31 | unknown |

*Characterized Proteins highlighted in Yellow and Putative Proteins highlighted in Cyan
