# Supplementary material for: Complex Carbohydrate Utilization by the Healthy Human Microbiome
Source: PLoS One. 2012 Jun 13;7(6):e28742. doi: 10.1371/journal.pone.0028742 (PMC3374616; doi:10.1371/journal.pone.0028742)
Supplement: Table S3 — Bacterial Genomes by Body Site. (DOCX) [file pone.0028742.s004.docx]

Table S3. Bacterial Genomes by Body Site.

| Bacterial Family | Eye | Blood UG | Lymph-nodes | GI Skin | Liver | GI UG | Blood Oral | Ear | Brain | GI Oral | Air Blood | Air Skin | GI | Skin | Oral Blood | Air | Bladder | Blood | Oral | UG |
| --- | --- | --- | --- | --- | --- | --- | --- | --- | --- | --- | --- | --- | --- | --- | --- | --- | --- | --- | --- | --- |
| *Actinomycetaceae* | 0 | 0 | 0 | 0 | 0 | 0 | 1 | 0 | 0 | 0 | 0 | 0 | 0 | 0 | 0 | 1 | 0 | 0 | 2 | 4 |
| *Bacillaceae* | 0 | 0 | 0 | 0 | 0 | 0 | 0 | 0 | 0 | 0 | 0 | 0 | 2 | 3 | 0 | 0 | 0 | 4 | 1 | 1 |
| *Bacteroidaceae* | 0 | 0 | 0 | 0 | 0 | 0 | 0 | 0 | 0 | 0 | 0 | 0 | 24 | 0 | 0 | 0 | 0 | 0 | 0 | 0 |
| *Bifidobacteriaceae* | 0 | 0 | 0 | 0 | 0 | 0 | 0 | 0 | 0 | 0 | 0 | 0 | 18 | 0 | 0 | 0 | 0 | 0 | 3 | 2 |
| *Burkholderiaceae* | 0 | 0 | 0 | 0 | 0 | 0 | 0 | 0 | 1 | 0 | 0 | 0 | 0 | 0 | 0 | 1 | 0 | 1 | 0 | 0 |
| *Campylobacteraceae* | 0 | 0 | 0 | 0 | 0 | 0 | 0 | 0 | 0 | 0 | 0 | 0 | 5 | 0 | 0 | 0 | 0 | 2 | 2 | 0 |
| *Chlamydiaceae* | 3 | 0 | 0 | 0 | 0 | 0 | 0 | 0 | 0 | 0 | 0 | 0 | 0 | 0 | 0 | 2 | 0 | 0 | 0 | 2 |
| *Coriobacteriaceae* | 0 | 0 | 0 | 0 | 0 | 0 | 0 | 0 | 0 | 0 | 0 | 0 | 3 | 0 | 0 | 0 | 0 | 1 | 3 | 1 |
| *Corynebacteriaceae* | 0 | 1 | 0 | 0 | 0 | 0 | 0 | 0 | 0 | 0 | 0 | 0 | 1 | 2 | 0 | 3 | 1 | 0 | 1 | 7 |
| *Enterobacteriaceae* | 0 | 0 | 0 | 0 | 0 | 0 | 0 | 0 | 1 | 0 | 0 | 1 | 32 | 0 | 0 | 2 | 0 | 4 | 0 | 3 |
| *Lactobacillaceae* | 0 | 1 | 0 | 0 | 0 | 1 | 0 | 0 | 0 | 1 | 0 | 0 | 35 | 0 | 0 | 0 | 0 | 0 | 1 | 13 |
| *Moraxellaceae* | 0 | 0 | 0 | 1 | 0 | 0 | 0 | 0 | 0 | 0 | 0 | 0 | 0 | 9 | 0 | 2 | 0 | 2 | 0 | 1 |
| *Mycobacteriaceae* | 0 | 0 | 0 | 0 | 0 | 0 | 0 | 0 | 0 | 0 | 0 | 0 | 0 | 3 | 0 | 2 | 0 | 0 | 0 | 1 |
| *Neisseriaceae* | 0 | 0 | 0 | 0 | 0 | 0 | 0 | 0 | 1 | 0 | 0 | 0 | 1 | 0 | 0 | 4 | 0 | 5 | 3 | 4 |
| *Pasteurellaceae* | 0 | 0 | 0 | 0 | 0 | 0 | 0 | 4 | 0 | 0 | 0 | 0 | 1 | 1 | 0 | 5 | 0 | 1 | 3 | 0 |
| *Staphylococcaceae* | 0 | 0 | 0 | 0 | 0 | 0 | 0 | 0 | 0 | 0 | 0 | 0 | 0 | 10 | 0 | 5 | 0 | 2 | 0 | 2 |
| *Streptococcaceae* | 0 | 0 | 0 | 0 | 0 | 0 | 0 | 1 | 2 | 0 | 1 | 0 | 2 | 0 | 1 | 8 | 0 | 1 | 9 | 0 |
