# Supplementary material for: Complex Carbohydrate Utilization by the Healthy Human Microbiome
Source: PLoS One. 2012 Jun 13;7(6):e28742. doi: 10.1371/journal.pone.0028742 (PMC3374616; doi:10.1371/journal.pone.0028742)
Supplement: Table S4 — Statistics of CAZy Genes per Genomes by Bacterial Family. (DOCX) [file pone.0028742.s005.docx]

Table S4. Statistics of CAZy Genes per Genomes by Bacterial Family.

| Bacterial Family | Number of Genomes | GH±PL mean±sd | GT51±GT28 mean±sd |
| --- | --- | --- | --- |
| *Actinomycetaceae* | 8 | 32.8±17.6 | 3.6±1.2 |
| *Bacillaceae* | 11 | 25.4±3.6 | 9.5±0.7 |
| *Bacteroidaceae* | 24 | 205.7±92.8 | 4.4±1.4 |
| *Bartonellaceae* | 2 | 6.0±1.4 | 3.0±0.0 |
| *Bifidobacteriaceae* | 23 | 42.5±15.1 | 2.8±0.4 |
| *Burkholderiaceae* | 3 | 34.3±11.0 | 6.7±2.5 |
| *Campylobacteraceae* | 9 | 3.4±0.9 | 2.7±0.7 |
| *Carnobacteriaceae* | 2 | 29.0±24.0 | 6.0±2.8 |
| *Chlamydiaceae* | 7 | 3.3±1.3 | 1.1±0.4 |
| *Clostridiaceae* | 15 | 73.2±92.6 | 4.9±2.5 |
| *Clostridiales Family XI. Incertae Sedis* | 6 | 17.2±7.9 | 2.3±0.8 |
| *Coriobacteriaceae* | 8 | 11.8±15.0 | 3.2±1.0 |
| *Corynebacteriaceae* | 16 | 17.3±7.8 | 3.2±0.8 |
| *Desulfovibrionaceae* | 3 | 8.3±3.2 | 2.3±0.6 |
| *Enterobacteriaceae* | 43 | 44.1±18.9 | 5.0±1.0 |
| *Enterococcaceae* | 17 | 53.8±5.2 | 4.0±0.0 |
| *Erysipelotrichaceae* | 7 | 49.6±36.2 | 4.0±1.6 |
| *Eubacteriaceae* | 7 | 34.6±19.7 | 3.3±0.8 |
| *Flavobacteriaceae* | 3 | 55.7±5.5 | 3.7±0.6 |
| *Francisellaceae* | 3 | 13.7±1.5 | 1.0±0.0 |
| *Fusobacteriaceae* | 16 | 12.5±11.5 | 2.8±0.4 |
| *Helicobacteraceae* | 13 | 3.3±1.8 | 2.1±0.3 |
| *Lachnospiraceae* | 9 | 60.3±73.5 | 3.4±1.8 |
| *Lactobacillaceae* | 52 | 31.3±13.1 | 3.6±1.0 |
| *Listeriaceae* | 6 | 47.2±2.9 | 3.0±0.0 |
| *Micrococcaceae* | 3 | 9.3±1.2 | 2.0±0.0 |
| *Moraxellaceae* | 15 | 11.1±3.1 | 3.8±0.6 |
| *Mycobacteriaceae* | 6 | 21.3±10.6 | 3.0±0.0 |
| *Neisseriaceae* | 18 | 10.8±4.3 | 3.1±0.8 |
| *Nocardiaceae* | 2 | 29.0±12.7 | 4.5±0.7 |
| *Oxalobacteraceae* | 2 | 14.5±2.1 | 3.0±0.0 |
| *Pasteurellaceae* | 15 | 11.3±4.0 | 4.1±0.8 |
| *Porphyromonadaceae* | 3 | 46.3±41.3 | 3.0±1.7 |
| *Prevotellaceae* | 10 | 76.9±38.4 | 4.8±1.7 |
| *Propionibacteriaceae* | 2 | 39.5±0.7 | 3.0±0.0 |
| *Pseudomonadaceae* | 2 | 32.0±1.4 | 4.5±0.7 |
| *Rikenellaceae* | 2 | 40.5±43.1 | 2.5±0.7 |
| *Ruminococcaceae* | 8 | 54.6±35.0 | 2.8±2.1 |
| *Sphingobacteriaceae* | 2 | 92.5±0.7 | 5.5±0.7 |
| *Spirochaetaceae* | 2 | 14.5±12.0 | 3.5±2.1 |
| *Staphylococcaceae* | 19 | 16.2±5.9 | 5.9±2.2 |
| *Streptococcaceae* | 25 | 33.5±8.6 | 4.1±0.4 |
| *Synergistaceae* | 2 | 8.5±3.5 | 4.5±2.1 |
| *Veillonellaceae* | 11 | 13.4±14.8 | 4.7±1.7 |
| *Vibrionaceae* | 4 | 46.2±18.5 | 4.2±2.1 |
