# Supplementary material for: Complex Carbohydrate Utilization by the Healthy Human Microbiome
Source: PLoS One. 2012 Jun 13;7(6):e28742. doi: 10.1371/journal.pone.0028742 (PMC3374616; doi:10.1371/journal.pone.0028742)
Supplement: Table S5 — Metagenomic Samples by Body Site. (DOCX) [file pone.0028742.s006.docx]

Table S5. Metagenomic Samples by Body Site.

| Body Sites | Number of Samples | Number of Samples with DOC | GH+PL  Mean Relative Abundance |
| --- | --- | --- | --- |
| Subgingival plaque | 8 | 6 | 7.84e+04 |
| Supragingival plaque | 99 | 57 | 2.22e+05 |
| Tongue dorsum | 108 | 106 | 3.63e+05 |
| Buccal mucosa | 95 | 23 | 6.89e+04 |
| Throat | 7 | 5 | 7.85e+04 |
| Palatine tonsils | 6 | 5 | 9.48e+04 |
| Attached gingivae | 6 | 4 | 3.44e+05 |
| Saliva | 5 | 2 | 3.83e+04 |
| Hard palate | 1 | 1 | 3.67e+05 |
| Stool | 119 | 119 | 1.82e+06 |
| Mid vagina | 2 | 0 | 5.32e+03 |
| Vaginal introitus | 3 | 0 | 1.02e+04 |
| Posterior fornix | 41 | 2 | 3.03e+04 |
| Anterior nares | 58 | 1 | 3.80e+03 |
| R_Retroauricular crease | 15 | 0 | 1.64e+05 |
| L_Retroauricular crease | 7 | 0 | 1.67e+05 |
