# Supplementary material for: Complex Carbohydrate Utilization by the Healthy Human Microbiome
Source: PLoS One. 2012 Jun 13;7(6):e28742. doi: 10.1371/journal.pone.0028742 (PMC3374616; doi:10.1371/journal.pone.0028742)
Supplement: Table S6 — CAZymes with Statistically Significant Differences in Relative Abundant between Body Sites. (DOCX) [file pone.0028742.s007.docx]

Table S6. CAZymes with Statistically Significant Differences in Relative Abundant between Body Sites.

| CAZy Family | Airways | Skin | Vagina | Oral | Gut | P-Value by Pairwise Comparison |
| --- | --- | --- | --- | --- | --- | --- |
| GH2 | 33882.1 ± 8153.05 | 30112.0 ± 15972.55 | 37537.4 ± 11778.38 | 36316.8 ± 4172.60 | 52597.2 ± 7597.50 | air-gut:0.04; oral-gut:0.05 |
| GH29 | 11557.3 ± 2412.36 | 9491.8 ± 4125.35 | 11467.4 ± 3316.08 | 12206.8 ± 1282.17 | 16511.7 ± 2206.59 | air-vagina:0.02; vagina-gut:0.01; vagina-oral:0.00; vagina-skin:0.01 |
| GH42 | 1707.3 ± 441.10 | 1656.1 ± 984.46 | 2335.5 ± 737.58 | 1922.7 ± 232.60 | 2871.7 ± 443.80 | skin-gut:0.01; vagina-skin:0.01 |
| GH30 | 1628.4 ± 448.21 | 1433.9 ± 883.49 | 2124.7 ± 742.81 | 1833.0 ± 245.69 | 3088.3 ± 519.83 | air-gut:0.04; oral-gut:0.00; skin-gut:0.01; vagina-gut:0.02 |
| GH55 | 523.1 ± 200.39 | 2.1 ± 1.27 | 502.3 ± 195.41 | 492.4 ± 85.84 | 858.5 ± 179.41 | air-gut:0.05; oral-gut:0.03; oral-skin:0.05; skin-gut:0.00; vagina-skin:0.03 |
| GH64 | 4.8 ± 2.49 | 0.5 ± 0.48 | 4.3 ± 2.11 | 5.1 ± 1.11 | 8.2 ± 1.97 | skin-gut:0.01 |
| GH20 | 16920.4 ± 3497.47 | 13942.5 ± 6112.46 | 16544.3 ± 4881.14 | 17607.0 ± 1776.14 | 24616.2 ± 3213.33 | vagina-gut:0.03; vagina-oral:0.03 |
| GH52 | 0.0 ± 0.03 | 0.0 ± 0.00 | 0.0 ± 0.00 | 0.0 ± 0.01 | 0.1 ± 0.03 | air-skin:0.00; air-vagina:0.02; oral-skin:0.00; skin-gut:0.00; vagina-gut:0.00; vagina-oral:0.00 |
| PL4 | 2.0 ± 0.97 | 0.0 ± 0.00 | 1.0 ± 0.60 | 1.0 ± 0.28 | 1.9 ± 0.71 | air-skin:0.00; oral-skin:0.00; skin-gut:0.00; vagina-skin:0.00 |
| GH23 | 14201.9 ± 1803.06 | 10190.6 ± 2811.00 | 10517.2 ± 2111.25 | 14309.5 ± 898.00 | 17204.7 ± 1448.70 | air-vagina:0.01; vagina-oral:0.02; vagina-skin:0.04 |
| GH114 | 32.0 ± 5.90 | 25.9 ± 11.30 | 21.0 ± 5.30 | 26.7 ± 2.44 | 40.4 ± 5.81 | air-vagina:0.01; vagina-gut:0.00; vagina-oral:0.01; vagina-skin:0.04 |
| GH6 | 365.0 ± 100.10 | 342.0 ± 205.98 | 93.8 ± 41.12 | 329.0 ± 47.29 | 330.8 ± 74.11 | air-vagina:0.01; vagina-gut:0.01; vagina-oral:0.01 |
| GH78 | 6948.7 ± 2185.72 | 6424.4 ± 4272.05 | 10405.1 ± 3831.89 | 7475.9 ± 1107.28 | 12246.9 ± 2212.09 | air-gut:0.05; oral-gut:0.02; skin-gut:0.04 |
| GH3 | 19235.9 ± 4235.20 | 16116.2 ± 7762.25 | 18490.6 ± 5569.20 | 20141.9 ± 2156.94 | 29884.2 ± 4088.16 | air-skin:0.04; air-vagina:0.04; oral-skin:0.01; vagina-gut:0.01; vagina-oral:0.04; vagina-skin:0.00 |
| GH26 | 953.0 ± 263.62 | 868.3 ± 560.01 | 989.7 ± 340.00 | 1020.0 ± 148.61 | 1701.7 ± 294.55 | air-gut:0.02; oral-gut:0.01; vagina-gut:0.01 |
| GH53 | 997.0 ± 304.06 | 857.4 ± 568.91 | 908.4 ± 335.28 | 1144.6 ± 159.83 | 1822.5 ± 307.18 | air-gut:0.01; oral-gut:0.03; vagina-gut:0.02 |
| GH116 | 407.2 ± 147.06 | 121.2 ± 105.36 | 504.3 ± 192.80 | 542.1 ± 92.60 | 883.0 ± 185.35 | air-gut:0.02; oral-gut:0.03; skin-gut:0.01 |
| GH28 | 11437.3 ± 3623.71 | 12611.3 ± 8618.46 | 14506.6 ± 5407.02 | 12302.1 ± 1805.54 | 18168.2 ± 3267.84 | oral-gut:0.03 |
| GH31 | 10060.7 ± 2357.95 | 8240.8 ± 4325.60 | 11392.6 ± 3217.88 | 10817.1 ± 1159.48 | 16251.7 ± 2254.76 | air-skin:0.04; air-vagina:0.00; oral-skin:0.01; skin-gut:0.01; vagina-gut:0.01; vagina-oral:0.02; vagina-skin:0.00 |
| GH33 | 4394.3 ± 795.27 | 4817.9 ± 1898.62 | 3557.5 ± 985.55 | 4064.5 ± 353.75 | 5259.2 ± 626.41 | oral-gut:0.04; skin-gut:0.02; vagina-skin:0.01 |
| GH4 | 951.0 ± 199.40 | 1139.0 ± 534.80 | 817.7 ± 211.13 | 920.6 ± 95.59 | 1176.4 ± 170.88 | air-vagina:0.02; vagina-gut:0.00; vagina-oral:0.02; vagina-skin:0.00 |
| GH8 | 1049.0 ± 162.34 | 670.9 ± 245.37 | 1320.0 ± 264.67 | 1327.6 ± 111.99 | 1625.1 ± 188.43 | air-skin:0.02; air-vagina:0.03; oral-skin:0.01; skin-gut:0.01; vagina-gut:0.03; vagina-oral:0.04; vagina-skin:0.00 |
| GH77 | 5874.6 ± 738.09 | 4393.4 ± 1066.99 | 4641.3 ± 941.13 | 6201.7 ± 391.43 | 7912.1 ± 668.04 | vagina-gut:0.03; vagina-oral:0.01 |
| GH74 | 310.1 ± 118.42 | 5.0 ± 4.51 | 272.5 ± 108.66 | 295.5 ± 50.64 | 488.9 ± 99.56 | oral-gut:0.03; oral-skin:0.04; skin-gut:0.00; vagina-skin:0.03 |
| PL1 | 3794.4 ± 1268.81 | 3696.0 ± 2686.63 | 5124.1 ± 2018.17 | 3958.8 ± 625.95 | 6461.4 ± 1289.52 | air-gut:0.03; oral-gut:0.02 |
| GH15 | 822.9 ± 234.62 | 710.3 ± 435.59 | 998.0 ± 364.88 | 858.6 ± 118.01 | 1310.9 ± 221.91 | skin-gut:0.02 |
| PL17 | 46.0 ± 23.08 | 0.0 ± 0.05 | 122.7 ± 55.08 | 82.0 ± 17.82 | 140.8 ± 36.13 | air-skin:0.04; oral-skin:0.01; skin-gut:0.00; vagina-skin:0.00 |
| GH86 | 0.2 ± 0.14 | 0.3 ± 0.29 | 0.4 ± 0.24 | 2.2 ± 0.86 | 3.4 ± 1.79 | air-gut:0.05 |
| GH44 | 10.7 ± 5.67 | 0.0 ± 0.00 | 0.3 ± 0.18 | 7.9 ± 2.80 | 12.3 ± 5.41 | air-skin:0.00; oral-skin:0.00; skin-gut:0.00; vagina-gut:0.04; vagina-skin:0.00 |
| GH14 | 0.0 ± 0.02 | 0.0 ± 0.00 | 0.0 ± 0.00 | 0.0 ± 0.01 | 0.0 ± 0.02 | air-skin:0.00; oral-skin:0.00; skin-gut:0.00; vagina-gut:0.03; vagina-skin:0.02 |
| GH63 | 2009.8 ± 640.93 | 1483.2 ± 1101.28 | 2350.5 ± 836.50 | 2289.6 ± 332.48 | 3330.7 ± 581.84 | air-gut:0.01; oral-gut:0.01; skin-gut:0.04 |
| PL21 | 274.9 ± 138.52 | 0.2 ± 0.09 | 129.3 ± 76.31 | 237.4 ± 61.12 | 289.3 ± 98.95 | oral-skin:0.05; skin-gut:0.01; vagina-skin:0.02 |
| GH16 | 4239.6 ± 962.36 | 3732.2 ± 1981.51 | 3988.6 ± 1348.31 | 4254.6 ± 473.93 | 6208.4 ± 910.35 | air-vagina:0.01; vagina-gut:0.00; vagina-oral:0.01 |
| GH43 | 27302.6 ± 8443.31 | 27747.3 ± 18838.30 | 34940.9 ± 12817.30 | 30868.6 ± 4450.32 | 46052.3 ± 8056.88 | air-gut:0.03; oral-gut:0.03 |
| GH65 | 1563.7 ± 249.41 | 1321.2 ± 527.20 | 1185.4 ± 239.37 | 1415.2 ± 110.48 | 1832.5 ± 206.29 | air-gut:0.04; oral-gut:0.04; vagina-gut:0.01 |
| GH51 | 4738.1 ± 1335.98 | 4146.5 ± 2380.92 | 5456.7 ± 1941.48 | 5331.6 ± 720.75 | 8189.2 ± 1341.77 | vagina-gut:0.05; vagina-skin:0.03 |
| PL6 | 47.1 ± 26.77 | 87.2 ± 87.14 | 113.2 ± 49.60 | 86.1 ± 17.40 | 153.6 ± 37.46 | air-gut:0.02 |
| GH115 | 2355.3 ± 793.47 | 2631.1 ± 1823.86 | 3382.0 ± 1321.24 | 2715.7 ± 417.91 | 3987.3 ± 745.53 | air-gut:0.03; oral-gut:0.03 |
| GH9 | 2185.8 ± 764.95 | 587.8 ± 403.56 | 1715.8 ± 626.85 | 2025.9 ± 327.56 | 3243.2 ± 640.85 | oral-gut:0.03; skin-gut:0.01 |
| GH113 | 43.6 ± 16.64 | 8.5 ± 6.37 | 43.5 ± 17.91 | 51.7 ± 8.81 | 107.4 ± 24.40 | skin-gut:0.00 |
| GH46 | 1.3 ± 0.45 | 2.0 ± 0.80 | 0.2 ± 0.13 | 1.2 ± 0.35 | 1.2 ± 0.66 | vagina-skin:0.04 |
| GH94 | 790.0 ± 280.32 | 63.9 ± 44.19 | 639.6 ± 235.48 | 1121.0 ± 188.20 | 1985.9 ± 387.16 | air-gut:0.00; oral-gut:0.04; oral-skin:0.02; skin-gut:0.00; vagina-gut:0.03; vagina-skin:0.01 |
| GH10 | 2170.4 ± 707.39 | 2207.8 ± 1480.99 | 2880.2 ± 1158.42 | 2586.5 ± 396.74 | 4112.1 ± 782.30 | air-gut:0.01; oral-gut:0.04 |
| GH110 | 975.0 ± 234.36 | 1002.3 ± 574.29 | 953.2 ± 344.08 | 989.5 ± 115.75 | 1278.7 ± 193.15 | vagina-gut:0.01; vagina-oral:0.04 |
| PL2 | 0.0 ± 0.00 | 0.0 ± 0.00 | 0.0 ± 0.00 | 0.0 ± 0.00 | 0.0 ± 0.02 | air-skin:0.00; oral-skin:0.00; skin-gut:0.00; vagina-skin:0.00 |
| PL3 | 0.0 ± 0.01 | 0.0 ± 0.00 | 0.2 ± 0.06 | 0.1 ± 0.01 | 0.0 ± 0.01 | air-oral:0.04; air-skin:0.00; air-vagina:0.03; oral-skin:0.00; skin-gut:0.02; vagina-skin:0.00 |
| PL11 | 1910.6 ± 651.99 | 2166.9 ± 1506.99 | 2578.2 ± 1068.20 | 2167.6 ± 354.00 | 3223.1 ± 652.66 | air-gut:0.04 |
| GH117 | 24.3 ± 10.68 | 7.0 ± 6.28 | 73.8 ± 59.43 | 48.0 ± 14.32 | 180.9 ± 67.65 | air-gut:0.00; air-oral:0.05; skin-gut:0.02 |
| GH25 | 6788.2 ± 741.84 | 5558.3 ± 1308.28 | 7786.5 ± 1764.84 | 8138.9 ± 580.73 | 10150.0 ± 1070.38 | air-vagina:0.04; vagina-gut:0.03; vagina-skin:0.02 |
| GH58 | 0.1 ± 0.14 | 0.0 ± 0.05 | 0.0 ± 0.00 | 0.1 ± 0.06 | 0.1 ± 0.09 | vagina-gut:0.00; vagina-oral:0.00; vagina-skin:0.00 |
| GH35 | 5015.2 ± 876.76 | 4785.5 ± 1952.55 | 4449.0 ± 1087.75 | 5529.9 ± 456.39 | 7180.8 ± 789.22 | vagina-gut:0.05; vagina-oral:0.01 |
| GH59 | 23.9 ± 10.00 | 0.1 ± 0.10 | 21.9 ± 9.81 | 22.7 ± 5.42 | 39.8 ± 10.55 | skin-gut:0.00 |
| GH1 | 5805.0 ± 659.68 | 4801.0 ± 1327.61 | 5512.4 ± 1127.92 | 6786.0 ± 453.44 | 7580.6 ± 751.67 | vagina-gut:0.02; vagina-skin:0.02 |
| GH68 | 484.3 ± 80.13 | 276.5 ± 103.69 | 476.2 ± 101.06 | 505.8 ± 44.56 | 485.2 ± 70.11 | air-skin:0.05; oral-skin:0.02; vagina-skin:0.01 |
| PL12 | 1259.0 ± 436.60 | 899.7 ± 697.20 | 1528.7 ± 628.25 | 1282.1 ± 212.52 | 2065.5 ± 433.54 | oral-gut:0.03 |
| GH92 | 18188.3 ± 4634.12 | 16192.4 ± 9226.60 | 20466.9 ± 6826.82 | 18516.1 ± 2239.13 | 27811.3 ± 4289.63 | oral-gut:0.01; vagina-gut:0.01 |
| GH19 | 261.4 ± 56.47 | 233.2 ± 94.93 | 109.6 ± 55.88 | 251.3 ± 27.10 | 227.0 ± 42.21 | air-gut:0.03; air-vagina:0.00; oral-gut:0.02; vagina-gut:0.03; vagina-oral:0.00; vagina-skin:0.05 |
| GH82 | 0.0 ± 0.00 | 0.0 ± 0.00 | 0.0 ± 0.00 | 0.7 ± 0.41 | 0.7 ± 0.64 | air-gut:0.03; vagina-gut:0.01 |
| GH112 | 876.0 ± 207.69 | 960.8 ± 538.80 | 439.5 ± 87.05 | 758.1 ± 82.62 | 1041.6 ± 159.59 | vagina-skin:0.03 |
| GH50 | 833.6 ± 373.03 | 1105.4 ± 912.16 | 1473.6 ± 630.14 | 861.5 ± 177.63 | 1592.7 ± 388.25 | air-gut:0.03; oral-gut:0.02 |
| GH97 | 12751.2 ± 3240.80 | 10028.2 ± 5512.69 | 13747.4 ± 4499.43 | 13187.4 ± 1611.76 | 20036.1 ± 3063.45 | oral-gut:0.01; vagina-gut:0.02 |
| GH18 | 7413.4 ± 1710.21 | 7328.1 ± 3700.60 | 8414.4 ± 2881.37 | 7126.3 ± 860.61 | 11592.1 ± 1888.63 | air-skin:0.03; oral-skin:0.02; skin-gut:0.00; vagina-skin:0.01 |
| GH27 | 2888.2 ± 768.70 | 2801.1 ± 1642.13 | 3076.5 ± 1009.86 | 2937.3 ± 388.47 | 4398.5 ± 711.01 | oral-gut:0.04 |
| PL13 | 463.6 ± 156.86 | 339.4 ± 264.69 | 730.9 ± 302.64 | 503.3 ± 84.76 | 863.4 ± 181.34 | air-gut:0.04; oral-gut:0.01; skin-gut:0.04 |
| GH67 | 947.0 ± 303.11 | 672.1 ± 448.49 | 1116.6 ± 449.18 | 1065.0 ± 162.81 | 1560.3 ± 292.53 | air-gut:0.03; oral-gut:0.05; skin-gut:0.04 |
| GH57 | 3313.3 ± 562.70 | 2607.9 ± 914.25 | 2564.1 ± 666.22 | 3175.1 ± 287.13 | 4443.6 ± 519.39 | air-vagina:0.05; vagina-gut:0.01 |
| GH36 | 6549.3 ± 1558.83 | 6286.9 ± 3510.09 | 6967.8 ± 2191.04 | 7380.1 ± 840.09 | 10804.4 ± 1555.01 | vagina-gut:0.01; vagina-oral:0.05 |
| GH101 | 690.2 ± 190.25 | 1005.8 ± 512.18 | 272.8 ± 69.34 | 555.7 ± 69.05 | 597.2 ± 129.59 | air-gut:0.05; oral-gut:0.02; skin-gut:0.02; vagina-skin:0.03 |
| GH12 | 0.0 ± 0.02 | 0.0 ± 0.00 | 0.0 ± 0.04 | 0.0 ± 0.01 | 0.0 ± 0.02 | air-skin:0.00; oral-skin:0.00; skin-gut:0.02; vagina-skin:0.00 |
| GH91 | 334.9 ± 150.54 | 3.2 ± 1.48 | 438.8 ± 255.99 | 310.8 ± 73.16 | 549.9 ± 159.02 | skin-gut:0.03 |
| GH48 | 1.4 ± 0.72 | 0.0 ± 0.05 | 1.8 ± 0.92 | 1.3 ± 0.32 | 2.1 ± 0.61 | skin-gut:0.04 |
| GH106 | 2164.0 ± 697.51 | 2201.9 ± 1532.84 | 3044.0 ± 1121.56 | 2345.1 ± 353.33 | 3554.4 ± 643.97 | air-gut:0.05; oral-gut:0.03 |
| GH17 | 85.0 ± 29.16 | 110.8 ± 61.19 | 32.5 ± 14.92 | 105.1 ± 20.07 | 117.0 ± 32.99 | vagina-gut:0.05; vagina-oral:0.05 |
| GH105 | 8105.6 ± 2599.45 | 7838.1 ± 5321.67 | 10849.3 ± 4124.24 | 9125.1 ± 1368.38 | 13636.6 ± 2481.19 | air-gut:0.03; oral-gut:0.02 |
| GH88 | 5365.4 ± 1589.57 | 4623.1 ± 3064.31 | 6582.4 ± 2354.15 | 5616.8 ± 800.46 | 8862.5 ± 1543.32 | air-gut:0.02; oral-gut:0.00 |
| GH11 | 5.5 ± 3.18 | 0.2 ± 0.19 | 2.5 ± 1.26 | 4.9 ± 1.37 | 7.6 ± 2.52 | skin-gut:0.05 |
| GH89 | 2430.7 ± 785.21 | 2360.9 ± 1728.88 | 3013.6 ± 1130.68 | 2603.4 ± 397.83 | 4111.6 ± 758.61 | air-gut:0.02; oral-gut:0.01 |
| PL15 | 803.5 ± 295.93 | 624.3 ± 528.02 | 1549.3 ± 660.99 | 840.1 ± 155.47 | 1684.6 ± 392.19 | air-gut:0.02; oral-gut:0.01 |
| GH99 | 34.4 ± 13.21 | 17.9 ± 17.61 | 155.0 ± 99.39 | 75.3 ± 23.37 | 167.8 ± 51.20 | air-gut:0.03 |
| GH109 | 3309.6 ± 790.81 | 2270.0 ± 1090.14 | 3079.0 ± 981.32 | 3361.7 ± 406.77 | 4640.7 ± 703.97 | vagina-gut:0.02 |
| PL9 | 396.2 ± 140.79 | 253.8 ± 250.01 | 667.2 ± 307.86 | 543.9 ± 91.10 | 903.9 ± 183.94 | air-gut:0.00; oral-gut:0.02; skin-gut:0.02 |
